# Invasive Ductal Carcinoma of the Breast With an Uncommon Widespread Thick Hyperechoic Pattern: A Case Report

**DOI:** 10.7759/cureus.103491

**Published:** 2026-02-12

**Authors:** Goki Oshita, Shoji Oura

**Affiliations:** 1 Department of Surgery, Kishiwada Tokushukai Hospital, Kishiwada, JPN

**Keywords:** breast cancer, extensive fat infiltration, metastatic lymph node with internal high echoes, microvoids around cancer cell clusters, thick high echoes

## Abstract

A 77-year-old woman visited our hospital due to a palpable breast mass. Mammography only showed a focal asymmetric density. In addition to one normal-sized lymph node with a punctate hyperechoic pattern, ultrasound showed widespread, thick hyperechoic areas with indistinct margins and adjacent hypoechoic areas. The hypoechoic areas had both an irregular shape and focal internal punctate high echoes on grayscale ultrasound, as well as blood inflow on Doppler ultrasound. Magnetic resonance imaging (MRI) of the mass showed weak high signals on fat-suppressed T2-weighted images and slow initial enhancement followed by persistent enhancement on dynamic studies, highly suggesting the hyperechoic areas to have abundant fat components. Core needle biopsy pathologically showed cancer cells with fat invasion.

The patient, therefore, underwent mastectomy and axillary dissection. Postoperative pathological study showed cancer cells spreading in 25-mm sizes in the fat tissue, with microvoids around cancer cell clusters, and two metastatic lymph nodes. Immunostaining of the tumor showed estrogen receptor positivity (Allred score 7), progesterone receptor negativity, human epidermal growth factor receptor type 2 equivocality (fluorescence in situ hybridization (FISH) negative), and a Ki-67 labeling index of 35%.

The patient has been well on adjuvant endocrine therapy without any events for three years. A diagnostic physician should note that microvoids just around cancer cell clusters may make the internal echoes of the metastatic lymph node high, and widespread cancer infiltration into the fat tissue generates an uncommon, widespread, thick hyperechoic pattern.

## Introduction

Mammography plays a central role in the diagnosis of breast cancer. In addition, magnetic resonance imaging (MRI) can provide breast surgeons with various important imaging findings, such as ductal spread and minute daughter nodules, to effectively determine the optimal surgical option. Ultrasound, giving neither pain nor irradiation to patients, is another inexpensive and very useful diagnostic modality to clearly depict breast disorders using tomographic images and to provide diagnostic physicians with important findings useful for image diagnosis. In short, ultrasound can show the location, size, internal echoes, posterior echoes, margin status, depth/width ratios, and blood flow of breast diseases, leading to the accurate diagnosis of breast disorders [1].

Scirrhous-type breast carcinomas, i.e., the most common breast cancer subtype, pathologically have abundant fibrous components both at the mass margins and within the masses. Fibrous components generate various typical ultrasound image findings, such as obscured mass margins, attenuated posterior echoes, low internal echoes, and a high tumor depth/width ratio [2]. For example, spiculated masses generally show these ultrasound images and have abundant fibrous components within and around the masses.

Various ultrasound findings mentioned above can contribute to the image diagnosis of breast disorders. Internal echo patterns naturally play important roles in the ultrasound diagnosis of the target lesions. The vast majority of breast specialists know that mucinous breast cancer often has a hyperechoic pattern, and malignant breast lymphoma generally shows an extremely hypoechoic pattern, based on their medical knowledge and clinical experience [3-5]. Very few diagnostic physicians, however, understand why mucinous breast cancer and malignant breast lymphoma have these patterns, respectively. It, therefore, provides great benefit to many clinicians, for accurate ultrasound diagnosis, to understand the hypoechoic and hyperechoic pattern formation mechanisms on ultrasound.

In this report, we present an invasive ductal carcinoma of the breast, which had an uncommon, widespread, thick hyperechoic pattern and metastatic axillary lymph nodes with internal diffuse punctate high echoes.

## Case presentation

**Figure 1:**
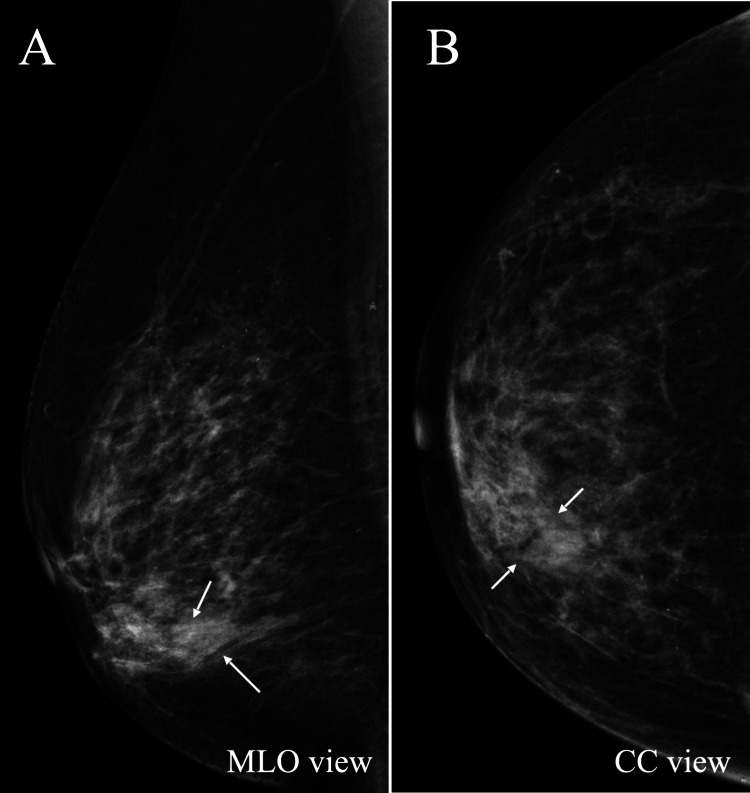
Mammography findings Mammography only showed focal asymmetric density (arrows) in the inner (B), lower (A) quadrant of the right breast.

A 77-year-old woman visited our hospital after noticing a breast mass. Palpation clarified an induration in the inner and lower quadrant of her left breast. Mammography only showed focal asymmetric density in the area corresponding to the induration (Figure 1).

**Figure 2:**
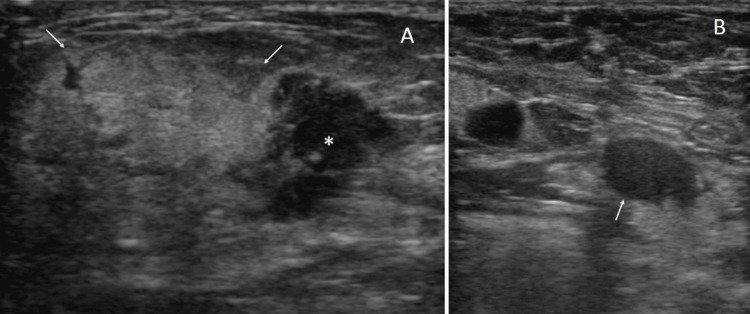
Ultrasound findings Ultrasound showed widespread, thick hyperechoic areas with indistinct margins (arrows), hypoechoic areas (asterisk) (A), and a lymph node with a diffuse punctate hyperechoic pattern (arrow) in the axilla (B).

Ultrasound showed widespread, thick hyperechoic areas, 31 mm in size, with indistinct margins and adjacent hypoechoic areas. The hypoechoic areas had irregular shapes, focal internal punctate high echoes, and internal vascularity. Ultrasound further showed no axillary lymph node swelling, but one lymph node, which had a diffuse punctate hyperechoic pattern (Figure 2).

**Figure 3:**
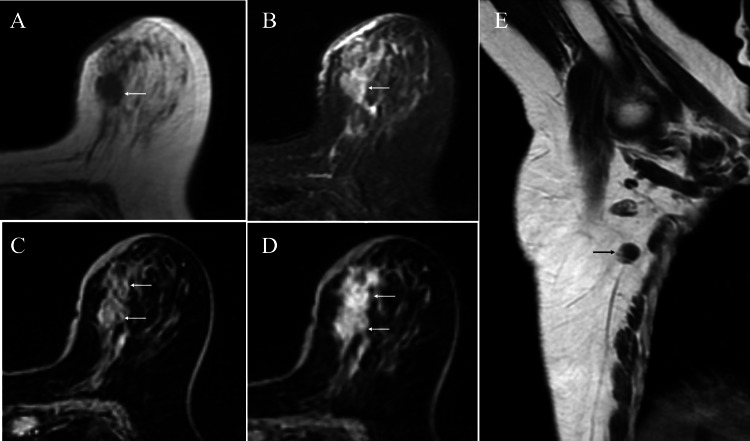
Magnetic resonance imaging (MRI) findings MRI of the tumor showed low signals on T1-weighted images (A, arrow), weak high signals on fat-suppressed T2-weighted images (B, arrow), and slow and persistent heterogeneous enhancement on the initial (C, arrows) and delayed (D, arrows) phase dynamic studies, respectively. Magnetic resonance axillography showed eccentric cortical hypertrophy (E, arrow) in one normal-sized lymph node.

MRI of the induration showed low signals on T1-weighted images, weak high signals on fat-suppressed T2-weighted images, and slow initial enhancement followed by persistent enhancement on dynamic studies. Magnetic resonance axillary imaging showed one non-enlarged lymph node with eccentric cortical hypertrophy (Figure 3).

**Figure 4:**
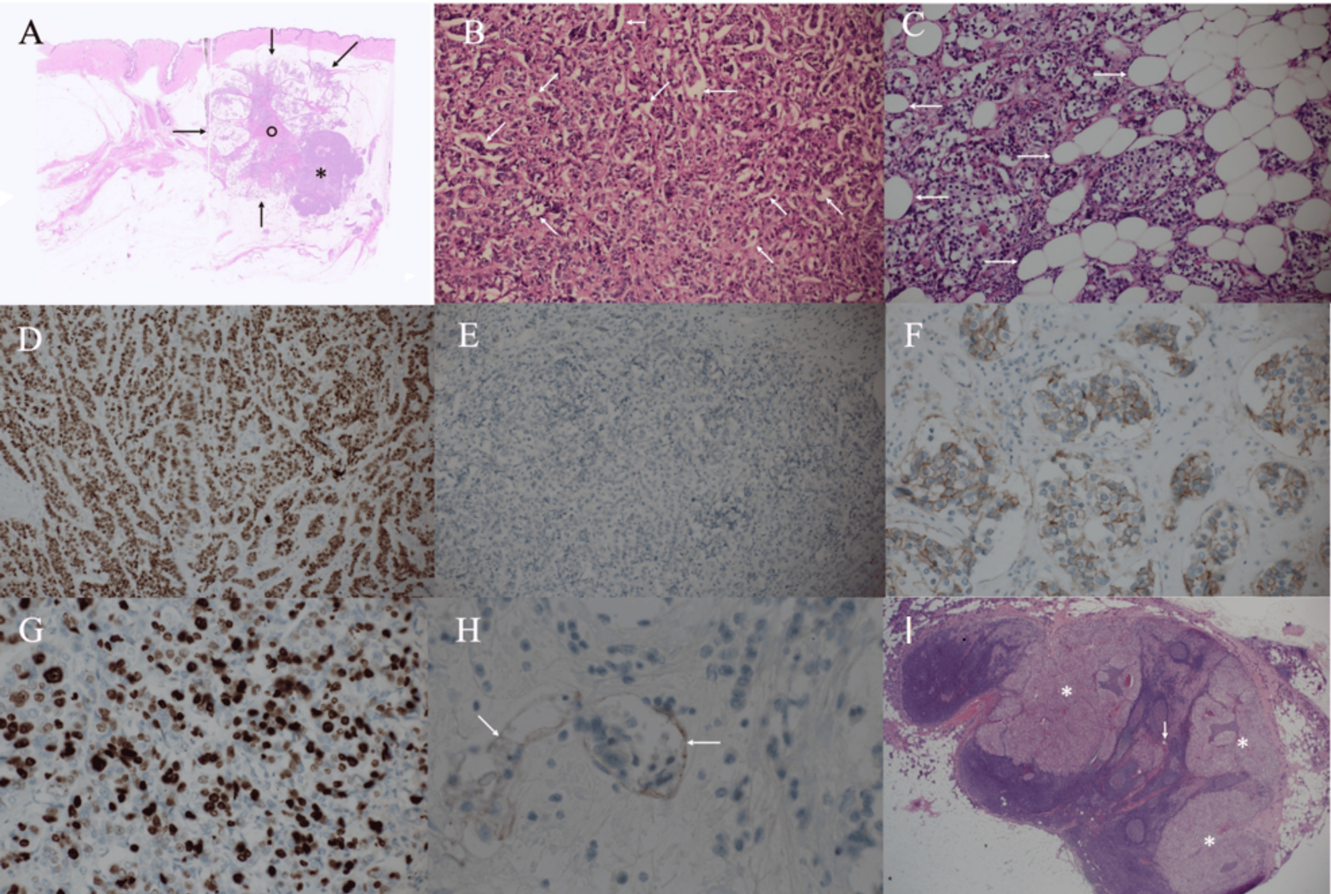
Pathological findings A) The tumor had solid areas (asterisk) and widespread cancer cells in the fat tissue (arrows), using the fibrous tissue as a stalk (open circle). B) Magnified view showed atypical cells densely growing, with microvoids (arrows) around cancer cell clusters. C) Magnified view showed cancer cell infiltration into the fat tissue (arrows). D) Immunostaining showed estrogen receptor positivity (Allred score 7). E) Immunostaining showed progesterone receptor negativity. F) Immunostaining showed human epidermal growth factor receptor type 2 equivocality. G) Immunostaining showed a Ki-67 labeling index of 35%. H) D2-40 immunostaining showed cancer cell involvement in the lymphatic vessels (arrows). I) Two normal-sized lymph nodes (arrow) had cancer cell metastasis (asterisks).

Core needle biopsy of the target areas in the breast pathologically showed atypical cells growing in a trabecular fashion with fat invasion. No malignant findings, however, were detected with aspiration biopsy cytology of the suspected metastatic lymph node in the axilla. Despite the absence of cytologically proven malignant cells in the possible metastatic lymph node, the patient strongly preferred axillary dissection without a sentinel lymph node biopsy after full discussion of the operative options. We, therefore, operated on the patient with a mastectomy and axillary dissection, with written informed consent about the operative option from the patient. Postoperative pathological study showed cancer cells growing in solid and trabecular fashions, spreading in 25-mm sizes, with microvoids around cancer cell clusters, marked fat invasion, and two metastatic lymph nodes. Immunostaining of the tumor showed estrogen receptor positivity (Allred score 7), progesterone receptor negativity, human epidermal growth factor receptor type 2 equivocality (fluorescence in situ hybridization (FISH) negative), and a Ki-67 labeling index of 35% (Figure 4).

The patient, unfortunately, developed partial skin flap necrosis, was discharged on the 10th day after surgery, and has been well for three years on adjuvant aromatase inhibitor therapy, without adjuvant chemotherapy, due both to her old age and the patient’s preference.

## Discussion

It is basic knowledge for breast specialists and diagnostic physicians that mucinous carcinoma of the breast often has a hyperechoic pattern [3]. In addition, lipid-rich carcinoma, invasive carcinoma with prominent necrosis, and fat necrosis can also have a hyperechoic pattern. Lipid-rich carcinoma presumably has very similar imaging findings to this case due to the similarity of pathological components constituting breast cancer, making it difficult to differentiate from this type of breast cancer. We can easily differentiate the latter two disorders from this type of breast cancer using MRI findings. Diagnostic physicians, therefore, tend to diagnose breast masses with a hyperechoic pattern as mucinous carcinoma due to its high prevalence among breast disorders. Diagnostic physicians can improve their diagnostic capabilities not only by knowing that breast mucinous carcinoma has a hyperechoic pattern, but also by understanding the mechanisms of hyperechoic pattern formation [6,7].

Invasive ductal carcinomas often have band-like high echoes, so-called haloes, just on the irregular mass, generally with a hypoechoic pattern, which implies cancer cell infiltration into the fat tissue, i.e., Category 5 [8]. Many diagnostic physicians also know these imaging and pathological findings, but do not fully understand the mechanisms that cause halo formation.

The same mechanisms generate a hyperechoic pattern in mucinous breast carcinoma and band-like hyperechoic areas around breast cancer. In other words, it is well known that ultrasound waves hit and backscatter against scattering bodies, forming the hyperechoic pattern depending on the degree of ultrasound backscattering [9]. Fat tissue, which has the least acoustic impedance in the body, makes cancer cells - when present in the fat tissue - act as scattering bodies, strongly generating ultrasound wave backscattering expressed as haloes on ultrasound. It is also well known that the hyperechoic pattern becomes brighter when the difference in acoustic impedance between the substances in contact within the mass increases.

In this case, cancer cells, with the fibrous tissue as a stalk, had infiltrated extensively into the fat tissue around the breast cancer, resulting in very specific ultrasound findings. In addition, the vast majority of cancer cell clusters have microvoids around them [10]. It is well known that microvoid-containing structures, such as tubular and papillary structures, can generate ultrasound wave backscattering and make the internal echoes of the target lesion high. It, therefore, is highly likely that the hyperechoic pattern in the normal-sized metastatic lymph node observed in this case was due to the numerous microvoids present around the cancer cell clusters. This image finding has the potential to help breast surgeons avoid unnecessary sentinel lymph node biopsies. On the other hand, the brightness of the hyperechoic pattern in the breast was clearly higher than that in the metastatic lymph nodes and was equivalent to the brightness seen in the haloes. We, therefore, speculate that the thick hyperechoic pattern was due more to the intermingling of adipocytes and cancer cells than to the presence of microvoids surrounding cancer cell clusters.

MRI can provide useful information for differentiating between invasive ductal carcinoma with the thick hyperechoic pattern and mucinous carcinoma. It is well known that mucinous carcinoma of the breast often presents high signals on T1-weighted images [11]. In addition, fat-suppressed T2-weighted images present high signals in mucinous carcinoma and weak high signals in invasive ductal carcinoma with marked cancer cell infiltration into the fat tissue. In any case, it is very important for diagnostic physicians to keep in mind that these rare cases exist and, when diagnosing lesions with similar ultrasound findings, to additionally evaluate them with MRI for accurate preoperative imaging diagnosis.

Diagnostic physicians generally focus on hypoechoic lesions in the diagnosis of breast diseases and tend to evaluate hyperechoic lesions as benign diseases or favorable mucinous carcinomas when making an ultrasound diagnosis. However, severe cancer cell infiltration into the fat tissue itself suggests aggressive tumor characteristics. In fact, this case had lymph node metastasis and a high Ki-67 labeling index of 35%. Diagnostic physicians, therefore, should speculate on and properly evaluate which pathological findings have generated the hyperechoic pattern in the target breast cancer, leading to the feasible selection of operative options and optimal surgical procedures.

## Conclusions

We experienced a breast cancer that had marked cancer cell infiltration into the fat tissue, and therefore showed the characteristic widespread, thick hyperechoic pattern. It naturally remains uncertain why cancer cells infiltrated the fat tissue so extensively in this case. A diagnostic physician should note that microvoids just around cancer cell clusters may make the internal echoes of the metastatic lymph node high, and that widespread cancer infiltration into the fat tissue generates the thick hyperechoic pattern. In addition, physicians should further be aware that lipid-rich carcinoma may present with similar imaging findings to this case, due to the similarity of the pathological components constituting the mass.

## References

[1] Kitano Y, Oura S, Honda M (2025). Accurate pathological prediction of small breast cancer with pathological component-based image evaluation: a case report. Cureus.

[2] Uozumi N, Oura S (2025). Mastopathic type breast fibroadenomas can have indistinct margins on ultrasound: a case report. Cureus.

[3] Takahashi H, Oura S (2025). Retained rim enhancement in mucinous breast cancer suggests aggressive biology: a case report. Cureus.

[4] Tomita M, Oura S, Nishiguchi H, Makimoto S (2020). A case of bilateral methotrexate-associated diffuse large B-cell lymphomas of the breasts with unique clinical presentation and outcome. Breast Cancer.

[5] Tomita M, Oura S, Nishiguchi H, Makimoto S (2020). A case of diffuse large B cell lymphoma of the breast with predominantly high-level internal echoes. Case Rep Oncol.

[6] Ohnishi J, Oura S, Shintani H (2025). The prediction of rare clear cell carcinomas of the liver via pathological component-based image evaluation: a case report. Cureus.

[7] Koyama A, Oura S (2025). Correlation of internal echo findings with the aggressiveness of diffuse large B-cell lymphomas: a case report. Cureus.

[8] Liberman L, Abramson AF, Squires FB, Glassman JR, Morris EA, Dershaw DD (1998). The breast imaging reporting and data system: positive predictive value of mammographic features and final assessment categories. AJR Am J Roentgenol.

[9] Yamamoto H, Oura S, Shintani H (2025). No mass image formation of metastatic neuroendocrine tumors to the liver on ultrasound: a case report. Radiol Case Rep.

[10] Takano Y, Oura S, Honda M (2025). Image findings of breast cancer-related elastosis: a case report. Case Rep Oncol.

[11] Zhang L, Jia N, Han L, Yang L, Xu W, Chen W (2015). Comparative analysis of imaging and pathology features of mucinous carcinoma of the breast. Clin Breast Cancer.

